# Molecular Typing and Antifungal Susceptibility of *Candida viswanathii,* India 

**DOI:** 10.3201/eid2410.180801

**Published:** 2018-10

**Authors:** Shamanth A. Shankarnarayan, Shivaprakash M. Rudramurthy, Arunaloke Chakrabarti, Dipika Shaw, Saikat Paul, Nandini Sethuraman, Harsimran Kaur, Anup K. Ghosh

**Affiliations:** Postgraduate Institute of Medical Education and Research, Chandigarh, India (S.A. Shankarnarayan, S.M. Rudramurthy, A. Chakrabarti, D. Shaw, S. Paul, H. Kaur, A.K. Ghosh);; Apollo Hospitals Enterprise, Chennai, India (N. Sethuraman)

**Keywords:** *Candida viswanathii*, fluconazole resistance, invasive candidiasis, emerging fungal infection, molecular typing, fungi, antimicrobial resistance, India

## Abstract

We report invasive candidiasis caused by *Candida viswanathii* over 2 time periods during 2013–2015 in a tertiary care hospital in Chandigarh, India. Molecular typing revealed multiple clusters of the isolates. We detected high MICs for fluconazole in the second time period.

Invasive candidiasis is a life-threatening infection caused by various *Candida* species. Although *C. albicans* has been the predominant species causing invasive candidiasis, non-albicans *Candida* (NAC) species have emerged globally (*1*). *C. viswanathii*, a pathogen first isolated from the cerebrospinal fluid of a patient in 1959 (*2*), is rarely encountered, and only 17 cases have been reported worldwide (*3*). This agent has been isolated sporadically from animal and environmental sources (*4*–*6*).

We report on 23 cases of invasive candidiasis caused by *C. viswanathii* at a tertiary care center in Chandigarh, India, involving 7 case-patients during December 2013–April 2014 and 16 case-patients during December 2014–April 2015. In the first time period, all isolates were from blood, whereas in the second time period, the agent was isolated from pus (n = 5), blood (n = 5), cerebrospinal fluid (n = 3), and lung nodule, lung aspirate, and iliac fluid (n = 1 each).

Of the 23 patients, 16 were men and 7 were women. Six (26%) patients had neutropenia, and 18 (90%) had tuberculosis, pancreatitis, or chronic kidney disease. Eight (34.7%) patients acquired the infection after surgery. Twelve patients used indwelling devices: 3 (15%) had a central venous catheter, 4 (20%) an endotracheal tube, 3 (15%) a drainage catheter, and 2 (10%) a urinary catheter.

We screened the hospital environment and the hands of healthcare workers for a possible source of *C. viswanathii* infection during the second time period. We could not isolate *C. viswanathii* from any of those samples from a total of 46 workers and 57 different environmental sites.

Conventional methods failed to differentiate *C. viswanathii* and *C. tropicalis*. *C. viswanathii* assimilated sucrose and cellobiose but failed to assimilate trehalose and raffinose. *C. tropicalis* has a variable assimilation pattern for these sugars.

To identify the isolates, we performed matrix-assisted laser desorption/ionization time-of-flight mass spectroscopy using MALDI-TOF MS, version 3 (﻿Bruker Daltonik GmbH, Bremen, Germany) and sequenced the D1/D2 region of a large subunit of ribosomal DNA (*7*,*8*). Because we could not identify *C. viswanathii* using the MALDI-TOF MS version 3 database, we updated the database in-house by adding sequence-proved isolates of *C. viswanathii*. We identified the isolates with a log score of >1.8 by using the modified database. The rDNA sequence of the isolates showed 100% similarity with the type strain of *C. viswanathii* ATCC 22981 (GenBank accession no. NG_054835.1) except for 1 isolate (99% similarity with type strain, accession no. MF682371). The molecular phylogenetic analysis revealed that 1 isolate (B-30815) had 1 nucleotide substitution (T to C), which was 1 of the 5 substitutions we observed in *C. pseudoviswanathii* while comparing it with *C. viswanathii* (*9*).

Amplified fragment-length polymorphism revealed a similarity coefficient of >90% of the isolates (Technical Appendix Figure) (*7*). The isolates from the first time period formed 2 clusters (cluster A and B); 1 isolate from the second period was also in cluster B. Isolates of the second time period had 3 major clusters (clusters C, D, and E) and had higher MICs for fluconazole.

We performed antifungal susceptibility testing for amphotericin B, fluconazole, itraconazole, voriconazole, posaconazole, caspofungin, anidulafungin, and micafungin by the microbroth dilution method recommended by the Clinical and Laboratory Standards Institute (*10*). After incubating the plates for 24 h at 37°C, we took a visual reading to determine the MICs. The isolates of the second time period had higher MICs (MIC_50_ 64 μg/mL, MIC_90_ 64 μg/mL) for fluconazole compared with the isolates of the first period (MIC_50_ 1 μg/mL, MIC_90_ 1 μg/mL). We also recorded higher MICs (MIC_50_ 1 μg/mL, MIC_90_ 2 μg/mL) for voriconazole for the isolates of the second period (Table).

**Table:** In vitro antifungal susceptibility data of *Candida viswanathii* isolates from a tertiary care hospital in Chandigarh, India

Isolate	Antifungal agent	MIC, μg/mL			
		Range	MIC _50_	MIC _90_	Geometric mean
*C. viswanathii* 2013–2014, n = 7	Amphotericin B	0.03–0.25	0.25	0.25	0.16
	Itraconazole	0.03–0.5	0.125	0.25	0.09
	Fluconazole	0.125–1	1	1	0.41
	Voriconazole	0.03–0.5	0.5	0.5	0.14
	Micafungin	0.125	0.125	0.125	0.125
	Caspofungin	0.5	0.5	0.5	0.5
	Anidulafungin	0.5–1	0.5	1	0.67
	Posaconazole	0.0312–0.0625	0.0312	0.0625	0.03
*C. viswanathii* 2015, n = 16	Amphotericin B	0.06–0.25	0.125	0.25	0.14
	Itraconazole	0.03–1	0.25	0.25	0.12
	Fluconazole	0.5–64	64	64	29.34
	Voriconazole	0.03–8	1	2	0.76
	Micafungin	0.125	0.125	0.125	0.12
	Caspofungin	0.125–1	0.5	0.5	0.40
	Anidulafungin	0.125–1	0.5	1	0.52
	Posaconazole	0.0321–0.5	0.25	0.25	0.17

In conclusion, our study showed multiple clusters of *C. viswanathii* causing invasive infections in patients with neutropenia and chronic diseases at a single healthcare center in India. We could not trace the source of the agent. Conventional identification methods could not differentiate the isolates from those of *C. tropicalis*. The high MICs for fluconazole among the isolates from the second time period also raise concerns about possible antifungal resistance. 

Technical Appendix Additional information about *Candida viswanathii* in a tertiary-care hospital in Chandigarh, India.
