# Supplementary material for: Molecular Typing and Antifungal Susceptibility of Candida viswanathii, India
Source: Emerg Infect Dis. 2018 Oct;24(10):1956–8. doi: 10.3201/eid2410.180801 (PMC6154138; doi:10.3201/eid2410.180801)
Supplement: Technical Appendix — Additional information about Candida viswanathii in a tertiary-care hospital in Chandigarh, India. [file 18-0801-Techapp-s1.pdf]

Article DOI: https://doi.org/10.3201/eid2410.180801

Molecular Typing and Antifungal Susceptibility of *Candida viswanathii*, India

Technical Appendix

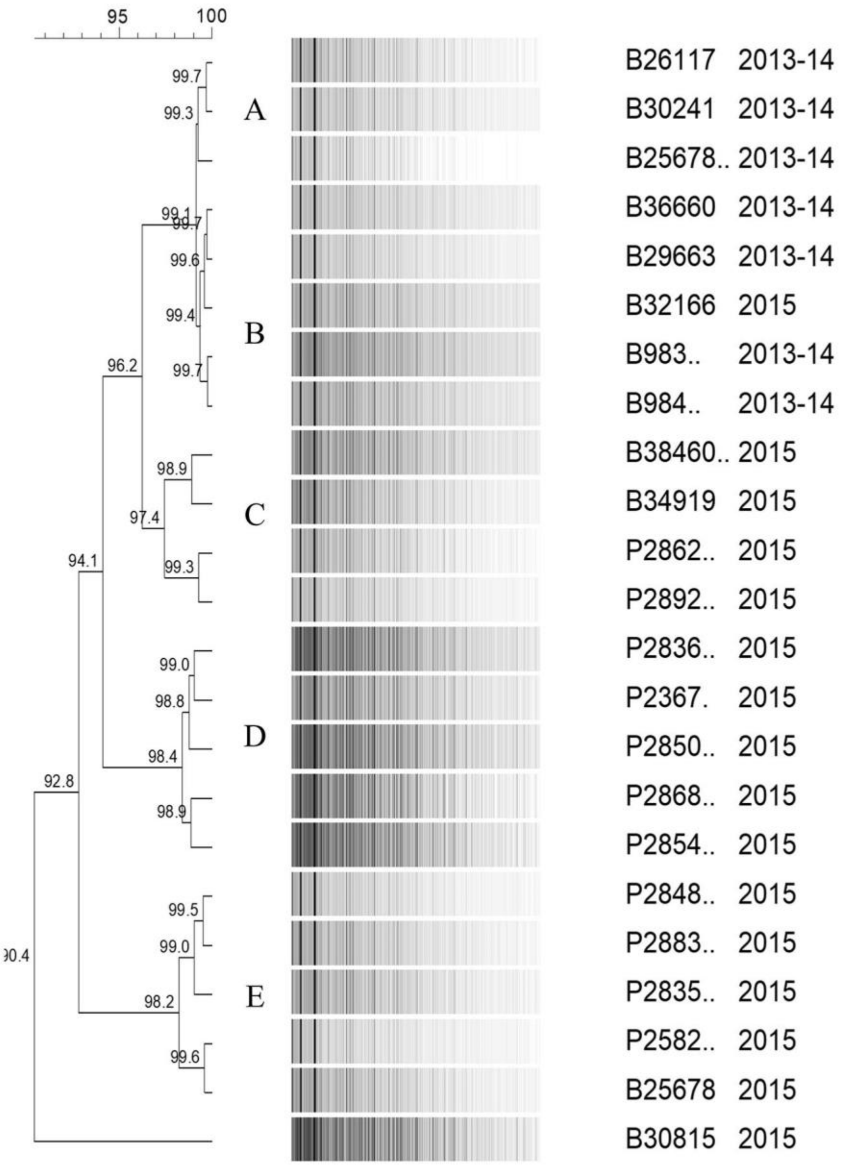

Technical Appendix Figure. Dendrogram generated by amplified fragment length polymorphism analysis of 23 *C. viswanathii* isolates revealing multiple clusters.
